# RK-33 inhibits the OC43 coronavirus and induces stress granules via DDX3X-independent mechanisms

**DOI:** 10.1261/rna.080931.125

**Published:** 2026-05

**Authors:** Cody J.S. Hecht, Roy R. Parker

**Affiliations:** 1 Department of Biochemistry, University of Colorado Boulder, Boulder, Colorado 80303, USA; 2 Howard Hughes Medical Institute, BioFrontiers Institute, University of Colorado Boulder, Boulder, Colorado 80303, USA

**Keywords:** DDX3X, integrated stress response, OC43, RK-33, stress granules

## Abstract

DDX3X is a human DEAD-box RNA helicase with multiple functions in RNA metabolism. Previous studies have suggested that DDX3X is an important proviral host factor for numerous RNA viruses, including HIV, HCV, and SARS-CoV-2, and may be targetable with inhibitors such as RK-33 for therapeutic benefit. In exploring the role of DDX3X and its homolog DDX3Y in coronavirus replication, we found that the DDX3X inhibitor RK-33 inhibits propagation of the OC43 coronavirus through a DDX3X/DDX3Y-independent mechanism. Knockdowns of DDX3X or DDX3X and DDX3Y had little effect on OC43 growth in multiple cell lines, yet RK-33 treatment reduced OC43 replication in the presence or absence of DDX3 proteins. We observed that RK-33 stimulates the integrated stress response independently of DDX3 proteins to cause stress granule formation, although this is not the primary mechanism by which RK-33 suppresses OC43. Together, our results show that DDX3 proteins are likely not general pro-coronaviral host factors, and caution should be used in interpreting results with RK-33 given its off-target activity.

## INTRODUCTION

The Covid-19 pandemic catapulted research into antiviral strategies against coronaviruses. Prophylactic vaccines and drugs targeting viral proteins have proven successful, but viral mutations can reduce their efficacy, necessitating continual updates to the therapy (Wang et al. 2021a; Liu et al. 2022; Stevens et al. 2022; Iketani et al. 2023). Targeting key host proteins that exert proviral activity during infection may overcome this limitation and provide broad-spectrum protection to future viral threats (Chitalia and Munawar 2020; Li et al. 2023). Dozens of host proteins that may participate in the life cycle of coronaviruses have been identified in genome-wide screens (Schneider et al. 2021; Wang et al. 2021b) and viral RNA and protein interactome studies (Gordon et al. 2020; Flynn et al. 2021; Hoffmann et al. 2021; Schmidt et al. 2021), but few have mechanistic support.

DDX3X is a DEAD-box RNA helicase involved in many areas of RNA metabolism, including transcription, splicing, nuclear export, and translation (Ariumi 2014; Mo et al. 2021). DDX3X emerged as a possible host-target for treatment of coronavirus infection when it was identified in three interactome studies, one for SARS-CoV-2 viral RNA (Schmidt et al. 2021) and two for SARS-CoV-2 nucleocapsid (Ciccosanti et al. 2021; Lodola et al. 2023). Subsequently, other groups have shown either proviral phenotypes (Vesuna et al. 2022) or little impact (Schmidt et al. 2021; Ariumi 2022) when DDX3X is depleted or inhibited during SARS-CoV-2 infection. Consensus is needed on whether DDX3X truly impacts coronavirus infection, and if so by what mechanism. Of note, DDX3X has a homolog on the Y chromosome, DDX3Y, which might complement some of DDX3X's function (Venkataramanan et al. 2021).

Several viruses interact with DDX3X during their life cycles. DDX3X has shown mostly proviral phenotypes during infection, though it can be antiviral in some cases (Schröder et al. 2008; Kesavardhana et al. 2021). The best characterized viral involvement of DDX3X is in HIV replication where it enhances nuclear export and translation of viral RNAs (Yedavalli et al. 2004; Soto-Rifo et al. 2012; Soto-Rifo et al. 2013). Roles for DDX3X with other viruses, in particular coronaviruses, largely remain unknown. Despite this, considerable effort has been placed into developing inhibitors for DDX3X that can be used as broad-spectrum antivirals (Brai et al. 2016; Kukhanova et al. 2020; Yang et al. 2020). These compounds are often used to study the function of DDX3X during infections and infer mechanisms from their phenotypes.

One commercially available DDX3X inhibitor, RK-33, was synthesized in 2015 (Bol et al. 2015) and has been used extensively to study the role of DDX3X in cancer (Bol et al. 2015; Xie et al. 2016; Winnard et al. 2024), viral infection (Yang et al. 2020; Ciccosanti et al. 2021; Vesuna et al. 2022; Mao et al. 2024), and stress granule regulation (Cui et al. 2020; Trussina et al. 2025; Zhu et al. 2025). RK-33 binds the Walker A motif of the DDX3X ATPase active-site at a *K*_D_ = 33 µM in ITC analysis, which is abolished with active site mutations (Yang et al. 2020). It has antiviral properties against coronaviruses and flaviviruses, though studies involving RK-33 have not converged upon mechanisms for DDX3X's role in the replication of these viruses.

In this study, we tested whether DDX3X/DDX3Y transient knockdowns or RK-33 suppressed the proliferation of the common cold coronavirus, OC43. We report that DDX3 proteins are not pro-OC43 factors and that RK-33 is antiviral toward OC43 independent of its activity against DDX3X. As part of its off-target activity, RK-33 activates the integrated stress response and induces stress granules, though these do not appear to be the off-target mechanisms by which RK-33 restricts OC43. These results suggest DDX3X is not a universal host factor for coronaviruses and reveal previously undescribed off-target effects of RK-33 on both viral replication and stress granule formation.

## RESULTS

### RK-33 inhibits OC43 growth independently of DDX3X

Given previously reported roles for DDX3X in coronavirus replication, we were interested in determining how DDX3X promoted coronaviral growth. We chose the nonpathogenic human coronavirus, OC43, as our model virus for use in BSL2 facilities. A previous study argued that DDX3X is an important pro-OC43 host factor in rhabdomyosarcoma (RD) cells, since the DDX3X inhibitor RK-33 inhibits OC43 replication (Vesuna et al. 2022). To validate these previous findings, we infected MRC-5 lung fibroblasts, which are commonly used to study OC43 replication (Uemura et al. 2021; Duncan et al. 2023; Martínez-Arribas et al. 2023), and treated them with RK-33.

**FIGURE 1.**
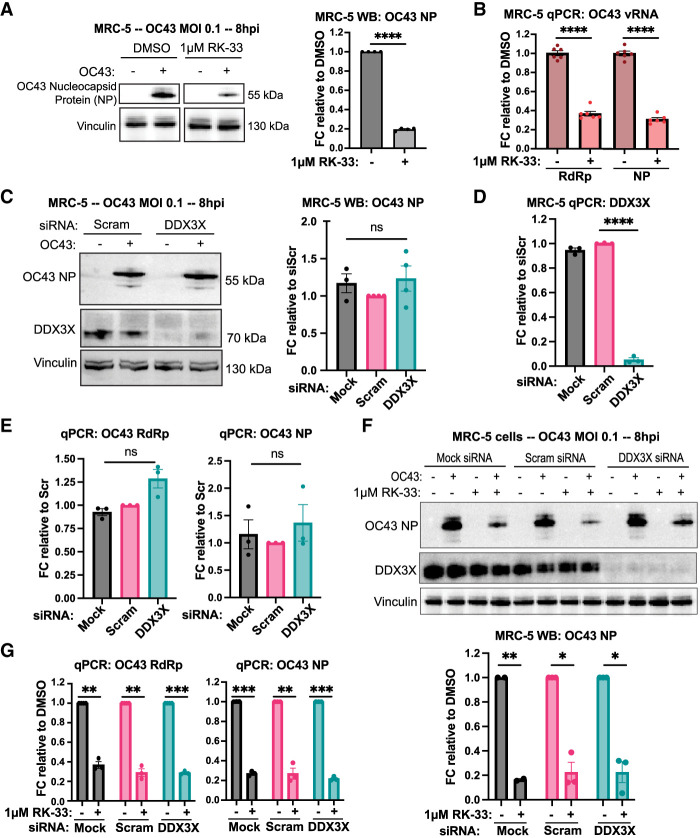
RK-33 treatment but not DDX3X knockdown suppresses OC43 growth. (*A*,*B*) MRC-5 cells were infected at an MOI of 0.1. Medium containing 1 μM RK-33 or DMSO was added to cells after 1 h of viral absorption, and cells were harvested and analyzed by WB (*A*) or qPCR (*B*) 8 h postinfection (hpi). Representative blot of four independent experiments is displayed. (*C*–*G*) MRC-5 cells were transfected with 20 nM siRNAs for ∼48 h, infected with OC43 with or without RK-33 for 8 hpi and analyzed as in *A* and *B*. Representative blots are shown from four (*B*) or three (*F*) independent experiments. qPCR quantification is made from at least three independent experiments. Statistical significance: (ns) not significant, (*) *P* ≤ 0.05, (**) *P* ≤ 0.01, (***) *P* ≤ 0.001, (****) *P* ≤ 0.0001. Tests used: one-sample *t*-test (*A*,*G*,*F*), two-sample *t*-test (*B*), one-sample and two-sample *t*-test (*C*,*D*,*E*). Error bars represent mean ± SEM.

We observed that 1 µM RK-33 treatment limited OC43 replication by about fivefold compared to the DMSO-treated controls when we probed the infected lysates for OC43-nucleocapsid protein (NP) by western blot (Fig. 1A). We also assessed the levels of genomic and subgenomic viral RNA levels during RK-33 treatment by performing qPCR for RNA corresponding to the OC43 *RdRp* and *NP* coding regions. We observed about a threefold decrease in gRNA and sgRNA abundance with RK-33 treatment compared to DMSO controls (Fig. 1B). These results were recapitulated in U2OS cells, where 2 µM RK-33 reduced OC43 NP protein levels by about half and *RdRp* and *NP* RNA levels to ∼40% of controls (Supplemental Fig. S1A,B). These results confirm that RK-33 limits OC43 replication, in accordance with previous studies.

To validate our intended pharmacological approach of DDX3X inhibition, we performed transient siRNA DDX3X knockdowns in MRC-5 cells. We achieved an almost complete knockdown as observed by the disappearance of DDX3X protein in WB staining and a ∼20-fold decrease in *DDX3X* RNA levels in qPCR (Fig. 1C,D). Surprisingly, DDX3X-deficient cells infected with OC43 showed little difference, if not a slight (∼1.2-fold) increase, in the NP protein or viral RNA levels compared to nontargeting controls (Fig. 1C,E). Similar experiments in U2OS cells revealed that DDX3X-knockdown elevated OC43 genomic and subgenomic RNA levels by approximately threefold compared to controls after 8 h of infection, suggesting an unexpected antiviral role for DDX3X in U2OS cells (Supplemental Fig. S1C,D). OC43 NP levels in U2OS cells were also elevated by roughly fourfold compared to controls after DDX3X knockdown, though this result was not statistically significant (Supplemental Fig. S1E). These results suggest that DDX3X is likely not a critical pro-OC43 factor and that RK-33 might exert antiviral activity independently of its inhibitory activity on DDX3X.

To ask if RK-33 suppressed OC43 independently of DDX3X, we treated DDX3X-knockdown MRC-5 cells with RK-33 and probed for NP in lysates by western blot. We observed a ∼80% reduction of OC43 NP levels in DDX3X-knockdown cells treated with RK-33 compared to knockdown cells alone, a decrease on par with control knockdown MRC-5 cells treated with RK-33 (Fig. 1F). Similarly, RK-33 treatment reduced OC43 RNA levels equally regardless of knockdown (Fig. 1G). We validated this result in U2OS cells and observed significantly reduced RNA and protein levels with RK-33 treatment irrespective of DDX3X knockdown (Supplemental Fig. S1E,F). This demonstrated that RK-33 exerts its antiviral activity in both the presence and absence of DDX3X.

**FIGURE 2.**
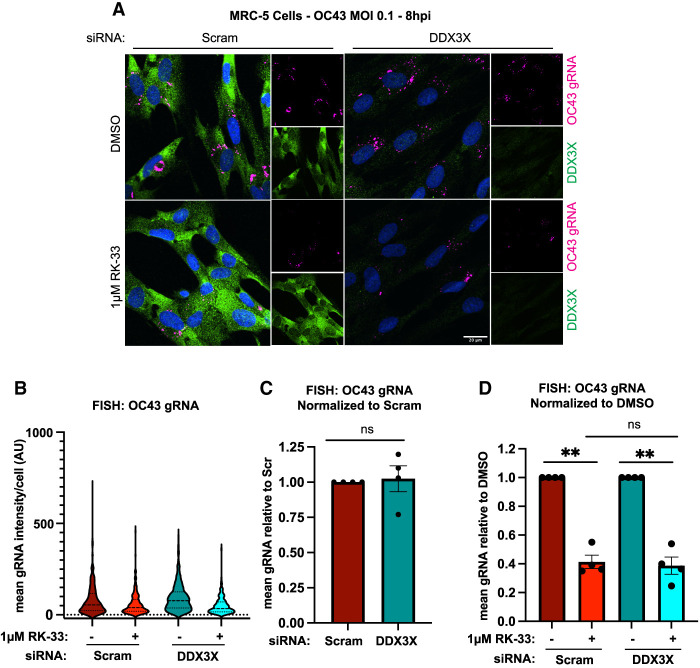
RK-33 suppresses OC43 replication independently of DDX3X. (*A*) MRC-5 cells were fixed after siRNA knockdown and OC43 infection as in Figure 1, and stained for OC43 genomic RNA by smFISH and DDX3X by IF. Representative images from four independent experiments are displayed. (*B*) Cells in *A* were segmented and within infected cells (OC43+); mean vRNA intensity was quantified on a single cell level. *N* > 45 cells/condition in each biological replicate from four independent experiments were analyzed. Representative analysis of replicate data is displayed. (*C*) The mean intensity of vRNA in OC43+ cells in *B* was normalized to Scramble knockdown levels across four independent experiments. (*D*) The mean intensity of vRNA in OC43+ cells in *B* was normalized to DMSO treatment across four independent replicates. Statistical significance: (ns) not significant, (*) *P* ≤ 0.05, (**) *P* ≤ 0.01, (***) *P* ≤ 0.001, (****) *P* ≤ 0.0001. Tests used: one- and two-sample *t*-test (*C*,*D*). Error bars represent mean and quartiles (*B*) or mean ± SEM (*C*,*D*).

To quantify differences in viral proliferation within individual cells, we fixed DDX3X-deficient or control MRC-5 cells 8 h after infection and stained for OC43 RNA by smFISH and DDX3X by immunofluorescence (Fig. 2A). IF staining and analysis confirmed successful DDX3X knockdown, with only a small percentage of cells displaying DDX3X expression (Fig. 2A; Supplemental Fig. S2A). We filtered and quantified OC43-positive cells (OC43+) by thresholding cells using viral smFISH signal. We observed little difference in the fraction of infected DDX3X-knockdown or control MRC-5 cells (Supplemental Fig. S2B) or the levels of viral RNA per OC43+ cell (Fig. 2B,C). Additionally, OC43+ DDX3X-deficient cells did not display any enrichment of DDX3X within the knockdown population (Supplemental Fig. S2C). These observations support the conclusion that DDX3X plays little role in OC43 replication.

However, in both DDX3X-deficient and control OC43+ cells, RK-33 treatment reduced the mean intensity of viral RNA by approximately threefold, in line with our qPCR data (Fig. 2B,D). Interestingly, RK-33 had a minimal effect (∼35%–40% decrease) on the fraction of infected cells in both DDX3X- and control-knockdown cells, despite the larger impact on viral RNA abundance (Supplemental Fig. S2D). These observations argue that RK-33 does not prevent viral entry or the export of newly produced virions, but rather induces a cellular state that is suppressive to viral proliferation. Together, these results establish that RK-33 remains antiviral even in DDX3X-knockdown cells and suppresses OC43 replication after cell entry.

DDX3Y is the Y-linked homolog of DDX3X and has shared (Venkataramanan et al. 2021) and distinct functions (Shen et al. 2022; Yanas et al. 2024). However, DDX3Y is rarely studied during viral infection. Since MRC-5 cells are derived from a male patient, we reasoned that DDX3Y could be compensating for DDX3X loss during siRNA experiments or be an equal target of RK-33, and thus responsible for our OC43 phenotype in these cells. To investigate the role of DDX3Y during infection, we transfected MRC-5 cells with DDX3Y siRNA alone or in combination with DDX3X siRNA and infected these cells with OC43. Notably, *DDX3Y* mRNA levels were ∼10-fold lower than *DDX3X* at baseline, suggesting that the *DDX3X* mRNA is the predominantly expressed transcript in these cells (Supplemental Fig. S3A). We assessed knockdown via qPCR with primers specific for *DDX3X* or *DDX3Y* and noted a ∼90%–95% and ∼70%–80% decrease in *DDX3X* and *DDX3Y* RNA levels, respectively, compared to nontargeting controls, confirming successful knockdowns (Supplemental Fig. S3B).

An important observation was that OC43 *RdRp* and *NP* RNA levels were unchanged across both single and double knockdown conditions, suggesting that DDX3Y does not play a major role during infection and does not compensate for DDX3X depletion in this context (Supplemental Fig. S3C). Moreover, RK-33 treatment consistently suppressed OC43 RNA levels in DDX3Y- and DDX3X/Y-depleted cells to a similar degree as controls (Supplemental Fig. S3D). We also validated these findings by fixing infected cells and staining for OC43 gRNA by smFISH (Supplemental Fig. S3E). We observed little difference in the mean cellular levels of viral RNA upon DDX3Y or DDX3X/Y knockdown compared to controls (Supplemental Fig. S3F). However, RK-33 treatment suppressed viral proliferation in DDX3Y- and DDX3X/Y knockdown cells, further supporting a DDX3-independent mechanism for RK-33 inhibiting viral growth (Supplemental Fig. S3F).

Together, these observations argue that DDX3 proteins have a limited role in supporting OC43 viral growth, contrary to previous suggestions of DDX3X as a pan-coronaviral host factor (Winnard et al. 2021; Vesuna et al. 2022). In addition, our data show that RK-33 has notable cellular activity independently of DDX3X, since DDX3-knockdown does not phenocopy RK-33 treatment during OC43 infection and OC43 remains sensitive to RK-33 in DDX3-depleted cells. Since these observations demonstrate that RK-33 alters cells independently of DDX3 proteins, we began exploring how RK-33 might affect cellular physiology independently of DDX3X.

### RK-33 induces the integrated stress response

Coronaviruses rely on host protein machinery to synthesize viral proteins necessary for infection. The integrated stress response (ISR) limits protein production under a range of environmental stressors, including viral infection, through phosphorylation of the key translation initiation factor eIF2α (Taniuchi et al. 2016). Given this, we examined if RK-33 treatment stimulates the ISR by probing RK-33-treated lysates for phosphorylated eIF2α and the transcription factor ATF4, whose translation is enhanced during the ISR.

**FIGURE 3.**
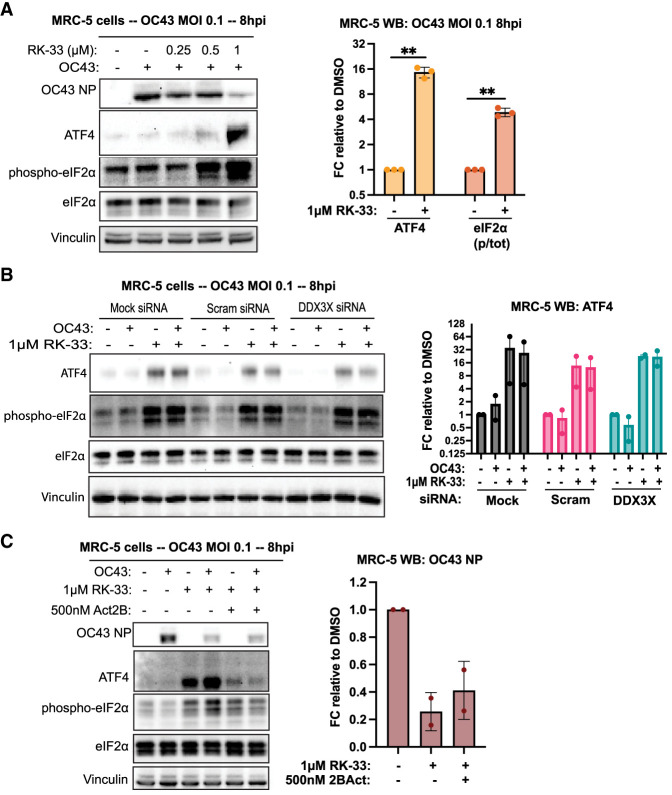
RK-33 activates the integrated stress response. (*A*) MRC-5 cells were titrated with RK-33 after viral absorption and probed for the indicated proteins at 8 hpi. Representative western blot shown. Quantifications were made from three independent experiments. (*B*) Transient siRNA knockdowns were performed as in Figure 1, followed by OC43 infection and WB analysis at 8 hpi. Representative western blot shown. Quantifications were made from two independent experiments. (*C*) Cells were pretreated ±500 nM 2BAct for 2 h, infected with OC43 (±2BAct treatment), and treated with RK-33 ±2BAct for the remainder of the infection period (8 h). Cells were lysed and analyzed by WB. Representative western blot shown. Quantifications were made from two independent experiments. Statistical significance: (ns) not significant, (*) *P* ≤ 0.05, (**) *P* ≤ 0.01, (***) *P* ≤ 0.001. Tests used: one-sample *t*-test (*A*). Error bars represent mean ± SEM.

We observed in MRC-5 cells that RK-33 induced phosphorylation of eIF2α in a dose-dependent manner, while ATF4 protein levels were substantially elevated at antiviral doses (>1 µM) (Fig. 3A). While OC43 infection alone had little impact on the ISR over the 8 h infection, 1 µM RK-33 treatment increased ATF4 and p-eIF2α protein levels by ∼15-fold and approximately fivefold, respectively. We observed a similar dose-dependent trend of RK-33 on p-eIF2a and ATF4 protein levels in U2OS cells (Supplemental Fig. S4A), with p-eIF2α and ATF4 both elevated by approximately sixfold compared to vehicle. These results indicate that RK-33 activates the ISR at the low-micromolar concentrations that inhibit OC43.

DDX3X has been shown to have a role in ATF4 translation during ISR induction in some contexts (Adjibade et al. 2017; Chen et al. 2025). To determine whether DDX3X is necessary for p-eIF2α and ATF4 upregulation during RK-33 treatment, we probed for these proteins in DDX3X-depleted MRC-5 cells. We observed a similar degree of ISR activation in DDX3X knockdown cells compared to controls, indicating that RK-33 stimulates the ISR in a DDX3X-independent manner (Fig. 3B). We validated DDX3X-independent increases in p-eIF2α and ATF4 upon RK-33 treatment in U2OS cells (Supplemental Fig. S4B). Induction of p-eIF2α by RK-33 was similar in DDX3X-deficient and control cells. However, while ATF4 protein levels were still elevated by RK-33 in DDX3X-deficient cells, the response was muted compared to controls, suggesting that DDX3X could have some role in ATF4 translation in U2OS cells. Together, these results show that RK-33 causes a cellular stress response that includes ISR activation and in a manner independent of OC43 infection and DDX3X inhibition.

If RK-33 stimulates an antiviral response via the ISR, one would expect that treatment with ISR inhibitors would abolish the growth phenotype we observe with OC43. ISRIB or its analog 2BAct maintain the eIF2B complex in an active state, effectively promoting eIF2 activity independently of its phosphorylation status (Wong et al. 2019). Therefore, we pretreated MRC-5 cells with 500 nM 2BAct, infected them with OC43, and assessed the ability of 2BAct to rescue OC43 growth during cotreatment with RK-33. While 2BAct was able to suppress ATF4 activation during 1 μM RK-33 cotreatment, it was unable to rescue OC43 growth (Fig. 3C). This observation argues that while RK-33 triggers the ISR, this is not the only, or primary, DDX3X-independent antiviral mechanism induced by RK-33.

### RK-33-induced stress granules assemble via activation of the ISR

**FIGURE 4.**
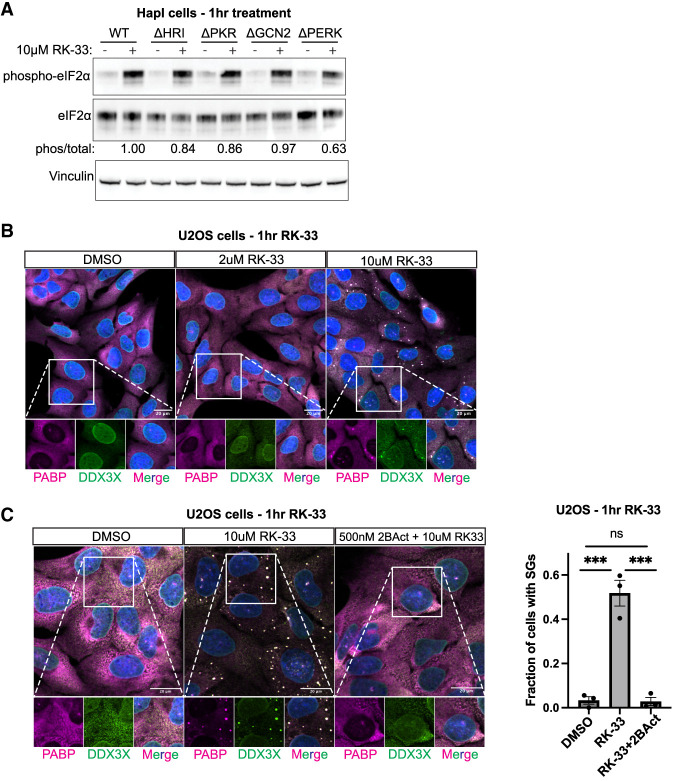
RK-33 activates multiple ISR kinases to induce stress granules. (*A*). HapI cells were treated with 10 μM RK-33 for 1 h before lysis and western blot for indicated proteins. Representative blot shown. (*B*) U2OS cells were treated with RK-33 for 1 h prior to fixation and immunofluorescence for DDX3X and PABP. Representative images shown, one independent experiment. (*C*) U2OS cells were pretreated with 2BAct for 1.5 h, followed by 10 μM RK-33 ±2Bact for 1 h prior to fixation and immunofluorescence for DDX3X and PABP. The percentage of cells with stress granules were determined. Representative image shown. *N* > 100 cells in each condition were quantified from three independent experiments. Statistical significance: (ns) not significant, (*) *P* ≤ 0.05, (**) *P* ≤ 0.01, (***) *P* ≤ 0.001. Tests used: unpaired *t*-test with Welch correction (*C*). Error bars represent mean ± SEM.

The ISR is activated by one of four kinases (HRI, PKR, GCN2, PERK) that phosphorylate eIF2α in response to diverse cellular stress (Taniuchi et al. 2016). To ask which of the ISR kinases phosphorylate eIF2α during RK-33 treatment, we treated HapI WT and HapI cells deficient for each of the kinases (ΔHRI, ΔPKR, ΔGCN2, ΔPERK) with RK-33 (Aulas et al. 2017). Surprisingly, each of the kinase-null cells was capable of phosphorylating eIF2α in response to acute RK-33 stress (Fig. 4A). Only ΔPERK was slightly diminished (∼60% of WT). This argues that no single kinase activates during RK-33 stress, and that RK-33 likely activates multiple eIF2α kinases.

RK-33 has previously been shown to induce stress granules (SGs) and slow the disassembly of SGs during stress resolution (Samir et al. 2019; Trussina et al. 2025; Zhu et al. 2025). Since RK-33 has been presumed to be a specific inhibitor for DDX3X ATPase function, the interpretation from these experiments has been that DDX3X plays an enzymatic role in limiting RNA–RNA interactions important for the structure and stability of RNP assemblies (Trussina et al. 2025; Zhu et al. 2025). Given our results that RK-33 activates the ISR, we examined if stress granule induction by RK-33 proceeds through the ISR, rather than DDX3X inhibition.

To test this, we treated U2OS cells with RK-33 at various concentrations and stained for DDX3X and the stress granule marker PABP by IF. We observed that 10 µM RK-33 induced stress granules in about half of cells during acute RK-33 stress, and that DDX3X colocalized with PABP in SGs (Fig. 4B), similar to previous observations (Valentin-Vega et al. 2016; Samir et al. 2019; Trussina et al. 2025). However, when we pretreated cells with 2BAct to blunt the ISR prior to RK-33 treatment, SGs were completely abolished (Fig. 4C). This demonstrates that stress granules induced by RK-33 activate through the ISR and further highlights that the off-target activity of RK-33 influences multiple aspects of cellular physiology.

## DISCUSSION

This work provides four conclusions. First, we have shown that the RNA helicase DDX3 proteins are not pro-OC43 host factors (Figs. 1, 2; Supplemental Figs. S1–S3). Second, the DDX3X inhibitor, RK-33, suppresses OC43 replication in the presence or absence of DDX3X and DDX3Y, demonstrating that RK-33 has biological impacts independent of its known targets (Figs. 1, 2; Supplemental Figs. S1–S3). Third, RK-33 treatment triggers the ISR, as part of its DDX3X-independent activity (Fig. 3; Supplemental Fig. S4). Fourth, the RK-33-induced ISR is responsible for SG assembly but not for OC43 viral suppression, suggesting that activation of the ISR is likely not the only off-target cellular alteration triggered by RK-33 (Figs. 3C, 4).

Our data demonstrate that DDX3X is not a pro-OC43 host factor, which is notable since other studies suggest DDX3X is broadly proviral during coronavirus infection (Winnard et al. 2021; Vesuna et al. 2022). While it is possible that a small percentage of DDX3X in siRNA-transfected cells can execute the requisite DDX3X functions during OC43 infection, the simplest explanation for a lack of phenotype in knockdown experiments is that DDX3X does not enhance OC43 infection, especially because high-DDX3X expressing cells are not enriched in OC43+ cells (Supplemental Fig. S2). Given previous studies suggesting a proviral role in SARS-CoV-2 infection (Ciccosanti et al. 2021; Vesuna et al. 2022), we speculate that DDX3X is a context-dependent host factor during β-coronavirus infection. Our data even show that DDX3X knockdown is contextual during OC43 infection in different cell types (U2OS vs. MRC-5). We found that DDX3X knockdown in U2OS cells trended with increased OC43 RNA and protein levels, while DDX3X knockdown in MRC-5 had little impact. Moreover, any compensatory effects of DDX3Y in the MRC-5 cell line are unlikely, since we observed no impact on viral replication upon DDX3Y knockdown or DDX3X/Y double KD, and RK-33 maintained OC43 suppression in these cells. Together, this argues that DDX3 proteins cannot be considered strictly pro-coronaviral and that DDX3X is not an ideal host-targeted factor for coronaviruses generally.

Our work highlights that RK-33 has multiple impacts on cells independent of its proposed inhibition of DDX3X. One of the off-target pathways activated by RK-33 is the ISR, which can have major influences on viral replication and regulation of cellular stress. Given this limitation, work inferring DDX3X function in viral infections solely based on RK-33 studies should be viewed with caution. Similarly, work using RK-33 to infer DDX3X function in stress granule regulation strictly as an RNA helicase should also be reconsidered (Trussina et al. 2025; Zhu et al. 2025). Our data show that RK-33 activates multiple ISR kinases to induce stress granules, which implies that RK-33 can cause several forms of cellular stress (i.e., ER + oxidative stress). Additionally, since ISR inhibitors are unable to reverse RK-33-mediated OC43 growth suppression, we conclude that RK-33 likely triggers additional off-target signaling pathways that may conflate any phenotypes strictly dependent upon the ability of RK-33 to inhibit DDX3X.

## MATERIALS AND METHODS

### Cell culture and viruses

MRC-5 cells were purchased from ATCC (CCL-171) and cultured in MEM (Thermo 11095098), supplemented with 1 mM sodium pyruvate, 1× NEAA (Thermo 11140050), and 10% FBS. Vero E6 and U2OS cells were gifts from Dr. Tony Schountz (CSU) and Dr. Paul Anderson (BWH) and were cultured in DMEM supplemented with 10% FBS. HapI cells were cultured in DMEM supplemented with 5% FBS. Infections were carried out in base media (MEM or DMEM) supplemented with 2% FBS (infection media).

### Drugs

RK-33 was ordered from Selleckchem (S8246), resuspended in DMSO to 10 mM, and stored in aliquots at −80°C. RK-33 was used between 100 nM and 10 µM in cell culture experiments. 2BAct was purchased from Cayman Chemicals (37788), resuspended in DMSO to 1 mM, and stored in aliquots at −80°C. 2BAct was used at 500 nM for cell culture experiments.

### Viral propagation and quantification

OC43 was propagated in Vero E6 cells according to the protocols of Fausto et al. (2023). Briefly, Vero E6 cells were seeded in T75 flasks at ∼2.5 × 10^6^ cells/flask. The next day, cells were washed 1× with infection media, and an MOI 0.01 inoculant of OC43 was added in 1 mL infection media. Flasks were rocked every 10–15 min for 1 h during viral absorption to ensure that all cells remained coated with the viral inoculant. After 1 h of virus exposure, cells were washed with infection media, and 12 mL of infection media was added to cells for 4–5 days at 33°C or until viral cytopathic effects were observed. Viral supernatants were harvested and centrifuged at 500*g* for 10 min at 4°C. Cleared supernatant was concentrated ∼10-fold through a 100 kDa Amicon filter (Sigma-Aldrich UFC9100) at 500*g*, 4°C. OC43 was stored in 250 µL aliquots at −80°C and quantified via limited dilution on Vero E6 cells by immunofluorescence of OC43 nucleocapsid protein (Sigma-Aldrich MAB9013), as previously described (Schirtzinger et al. 2022).

### Infection of human cells

WT MRC-5 cells were seeded at 4 × 10^5^ cells/well (6 well dish) or 2 × 10^5^ cells/well (12 well dish) or U2OS cells at 3.5 × 10^5^ cells/well (6 well dish) or 1.75 × 10^5^ cells/well (12 well dish). The next day, an OC43 inoculant of MOI 0.05–0.2 (MRC-5 cells and U2OS cells) was added to cells for 1 h with rocking every 10–15 min for viral absorption. Cells were washed 2× in infection media and incubated in infection media at 33°C for the indicated duration prior to analysis.

### siRNA knockdown

siRNAs were purchased from Horizon (ON-TARGETplus siRNA SMARTPools: DDX3X (1654) M-006874-01-0005; DDX3Y (8653) L-011904-01-0005; nontargeting siRNA #1 D-001810-01-05). MRC-5 cells were seeded at 3 × 10^5^ cells/well in a 6 well plate for transfection with siRNAs the next day. U2OS cells were reverse transfected with 5 × 10^5^ cells/well in a 6 well plate. Targeting and nontargeting siRNAs were complexed by Lipofectamine RNAiMAX (Thermo 13778075) according to the manufacturer's instructions and added to cells for a final concentration of 20 nM (2.5 mL volume). Mock transfection conditions include transfection reagent with no siRNA. After ∼36 h, cells were harvested, counted, and reseeded for infection the following day for ∼48 h of total knockdown. DDX3X/DDX3Y double knockdown was performed with 10 nM of each siRNA.

### Western blot

Cells were washed 1× with cold PBS and lysed with RIPA buffer (Thermo 89900) containing 1× protease and phosphatase inhibitors (CST 5872S). Soluble proteins were separated by centrifugation and separated on 4%–12% BIS-Tris PAGE gels. Proteins were transferred to PDVF or nitrocellulose membranes, blocked in TBS–Casein (Bio-Rad 1610782), and probed with primary antibodies overnight at 4°C. Membranes were washed in TBST and probed with HRP-conjugated anti-Mouse or anti-Rabbit secondary antibodies (CST) for 1 h at room temperature. Membranes were washed again and incubated with chemiluminescent substrate (Thermo 34096) before imaging. The following primary antibodies and dilutions were used for western blot: mouse anti-Vinculin (Sigma-Aldrich V9131, 1:10,000); mouse anti-OC43 nucleocapsid protein (Sigma-Aldrich MAB9013, 1:1000), mouse anti-DDX3X (Santa Cruz SC-365768, 1:500), rabbit anti-ATF4 (Cell Signaling Technology CST11815T, 1:1000), rabbit anti-eIF2α (Cell Signaling Technology CST9722S, 1:1000), rabbit anti-GAPDH (Cell Signaling Technology CST2118, 1:2000), and rabbit anti-phospho-eIF2α (Abcam ab32157, 1:1000). Secondary antibodies goat antimouse HRP (CST 7076S) and goat antirabbit HRP (CST 7074S) were used at 1:4000.

### qPCR

After treatment, cells were washed twice with PBS to remove extracellular viruses in the culture dish and lysed in TRIzol reagent (Thermo 15596018) and frozen at −80°C until processing. RNA was extracted with Zymo Direct-zol RNA Purification Kit (R2050) following the manufacturer's protocol, including on-column DNase I treatment. RNA was quantified with a NanoDrop 2000 (Thermo). Equal RNA amounts (500–1000 ng) were reverse transcribed using iScript RT (Bio-Rad 1708891) by following the manufacturer's protocols. Following reverse-transcription, cDNAs were diluted 1:10 in water, and frozen at −20°C until qPCR. qPCR was performed using SYBR Green PowerTrack (Thermo 4309155) using 2.5 ng of equivalent RNA, 125 nM primers, and 1× SYBR enzyme mix. Samples were amplified in C1000 Touch Thermal Cycler (Bio-Rad) with the following protocol: 1 step of 95°C for 2 min; 40 steps of 95°C for 15 sec and 60°C for 60 sec. Following qPCR, a melt was performed with a 15 sec denaturation at 95°C, followed by 60°C for 60 sec with a 1.6°C/sec ramp rate, followed by a ramp to 95°C with a 0.075°C/sec rate. *Ct* values were obtained using a single threshold with Bio-Rad CFX Manager Analysis (V3.1). 2^(−ΔΔ*CT*)^ analysis was performed using GAPDH as a reference, as no change in GAPDH expression was observed during OC43 infection time course experiments or RK-33 treatment. Relative comparisons of gene abundance were made as indicated in the figure panels, typically against scrambled siRNA treatment or DMSO treatment. qPCR primer sequences can be found in Supplemental Table S1. gDNA contamination and primer specificity were assessed by melt analysis and gel electrophoresis of uninfected and RT samples. qPCR efficiency for each primer was confirmed by a dilution series of cDNA.

### Generation of smiFISH probes

smiFISH probes complementary to positive-strand OC43 genomic RNA (NCBI Reference Sequence: NC_006213.1, Nsp3 coding region, bases 3000–5000) were designed using Biosearch Technologies Stellaris Probe Designer version 4.2. The FLAPY sequence (Tsanov et al. 2016) was appended to the 3′ end of each probe and ordered from IDT. The secondary FLAPY probe (ligated to ATTO550 or ATTO647n at both the 3′ and 5′ ends) was ordered from IDT. Twenty-four probes were combined at equimolar ratios (100 µM combined concentration), and annealed to the FLAPY secondary in a 50 µL reaction containing 1× NEB buffer 3, 5 µM combined OC43 probes, and 4 µM FLAPY secondary at 85°C for 3 min, 65°C for 3 min, and 25°C for 5 min. Annealed probes were stored at −20°C. Probe sequences can be found in Supplemental Table S1.

### Combined FISH + IF

Cells grown on glass coverslips or glass-bottom dishes were fixed in 4% PFA for 10 min at room temperature, washed three times with PBS, and permeabilized in 70% ethanol for at least 15 min at 4°C. Fixed and permeabilized cells were washed in 2× SSC + 10% formamide (wash A) before probing with 100 nM probe mix in hybridization buffer (10% formamide, 10% dextran sulfate, in 2× SSC) overnight at 37°C in a humidity chamber. The next day, coverslips were washed twice in 2× SSC and mounted onto a glass slide with ProLong Glass Antifade Mountant with NucBlue (Thermo P36983) for at least 3 h. For immunofluorescence with FISH, after probe hybridization, coverslips were washed twice in 2× SSC, three times in PBS and probed for primary antigen in PBS overnight at 4°C or at room temperature for 2 h. Coverslips were washed 5× in PBS, and secondary antibody in PBS was added for 2 h at RT. Cells were washed 5× in PBS and mounted with ProLong Glass Antifade Mountant with NucBlue. FISH probe sequences can be found in Supplemental Table S1. The following primary antibodies and dilutions were used for IF: mouse anti-DDX3X (SC-365768, 1:200), rabbit anti-PABP (Abcam ab21060, 1:1000). Secondary antibodies goat antimouse AF488 (Thermo A32723), goat antirabbit AF647 (Abcam ab150079), goat antimouse ATTO550 (Millipore 43394), and goat antirabbit ATTO488 (Millipore 18772) were all used at 1:1000 dilutions.

### Immunofluorescence without FISH

Cells grown on glass coverslips or glass-bottom dishes were fixed in 4% PFA for 10 min at room temperature, washed three times with PBS, and permeabilized in 0.2% Triton-X 100 for 15 min at 4°C. After five PBS washes, cells were blocked in 3% BSA in PBS for >1 h at room temperature, followed by primary antibody incubation overnight at 4°C in 3% BSA in PBS. In the morning, cells were washed in PBS five times and incubated with secondary antibody for 2 h at room temperature. Cells were counterstained in Hoechst 33342 (Thermo 62249) at 2 µM in PBS for 10 min prior to mounting with ProLong Glass Antifade Mountant with NucBlue (Thermo P36983).

### Microscopy

Imaging was performed at room temperature with the Nikon spinning disk super resolution by optical pixel reassignment (SoRa) microscope, with a 20× NA 0.75 air objective, 40× NA 1.15 water immersion objective, or 60× NA 1.27 water immersion objective, and a Hamamatsu ORCA Fusion BT sCMOS Camera.

### Microscopy analysis

Images were analyzed in Cell Profiler as single-slice frames. Cells were segmented on Hoechst and PABP staining for nuclei and cytoplasm, respectively. Infected cells were filtered by thresholding cytoplasmic OC43 gRNA signal, with biological replicates (imaged on different days with independent imaging parameters) analyzed individually. Thresholds were set at the intensity of single viral RNAs in each individual experiment, resulting in 1%–3% false positive infection rate in untreated controls due to probe artifacts. The fraction of infected cells was normalized to that of Scram knockdown or DMSO to compare biological replicates (Supplemental Fig. S2). For quantification of OC43 gRNA abundance, the mean cytoplasmic intensity of gRNA and DDX3X was determined in infected and uninfected cells. The mean cytoplasmic gRNA intensity from uninfected cells was subtracted from that of infected cells to account for the contribution of the staining background on mean intensity. Infected cells with mean gRNA intensity above background levels were analyzed to compare gRNA levels from individual infected cells within the population (Fig. 2B). Background-subtracted mean gRNA intensity was normalized to siScram or DMSO to compare biological replicates (Fig. 2C,D). Stress granules were identified as primary objects (5–15 pixels) in Cell Profiler from 60× single slice images after enhancing speckles (pixel size 15).

## DATA DEPOSITION

All data in this manuscript are available from the corresponding author upon request.

## SUPPLEMENTAL MATERIAL

Supplemental material is available for this article.

## COMPETING INTEREST STATEMENT

R.R.P. is a cofounder and consultant for Illumen Therapeutics and is on the scientific advisory board for Ascidians Therapeutics.

## Figures and Tables

**Cody J.S. Hecht:**
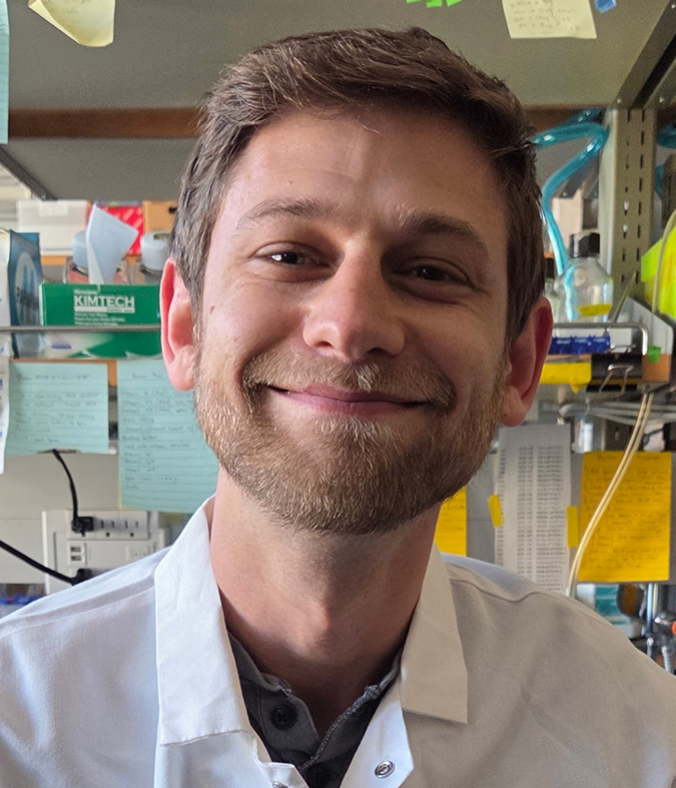


## MEET THE FIRST AUTHOR

***Meet the First Author(s)* is an editorial feature within *RNA*, in which the first author(s) of research-based papers in each issue have the opportunity to introduce themselves and their work to readers of *RNA* and the RNA research community. Cody J.S. Hecht is the first author of this paper, “RK-33 inhibits the OC43 coronavirus and induces stress granules via DDX3X-independent mechanisms.” Cody is a graduate student in the Biochemistry Department at the University of Colorado Boulder, where he studies how coronaviruses leverage host programs for their replication.**

**What are the major results described in your paper, and how do they impact this branch of the field?**

The main point of our study is that the widely used DDX3X inhibitor, RK-33, has significant impacts on cellular physiology independently of DDX3X. The two DDX3X-independent activities that we identified here are induction of stress granules via the integrated stress response and suppression of OC43 coronaviral growth. We hope that researchers studying the function of DDX3X in stress responses or viral infection will be cautious in interpreting results from RK-33 experiments, since these off-target activities will likely interfere with any phenotypes strictly dependent on DDX3X.

**What led you to study RNA or this aspect of RNA science?**

When I began my PhD research in the Parker lab, I became interested in understanding how host RNA-binding proteins interact with RNA viruses to enhance their proliferation during infection. Following the emergence of SARS-2, several groups proposed that RNA helicases such as DDX3X were important for SARS-2 to proliferate. These studies led us to investigate a potential role for DDX3X in OC43 infection.

**During the course of these experiments, were there any surprising results or particular difficulties that altered your thinking and subsequent focus?**

Absolutely—very early in the project, we observed the surprising result that RK-33 treatment and DDX3X-knockdown led to different phenotypes during OC43 infection. From there, we switched our focus from describing a function for DDX3X in coronaviral infection to describing the off-target activities of RK-33. It is a good example of beginning a set of experiments with an initial hypothesis, and having the data drive the project in a completely different direction.

**What are some of the landmark moments that provoked your interest in science or your development as a scientist?**

My first wet lab experience during an undergraduate summer at UMass Medical School was a defining moment for me. It exposed me to the thrill of research and the passion of thoughtful, creative scientists who inspired me. I joined a research lab at school that fall and have been in science ever since. I'm so grateful to Paul, Sangram, Aaron, and others in the Thompson lab who showed me how fun and rewarding research can be.

**If you were able to give one piece of advice to your younger self, what would that be?**

I would tell my younger self that progress comes in waves, understanding doesn't come overnight, and hard work pays off.
